# Maternal Morbidity and Complications in Hemolysis, Elevated Liver Enzymes, and Low Platelet Count (HELLP) Syndrome: A Retrospective Case Series

**DOI:** 10.7759/cureus.103532

**Published:** 2026-02-13

**Authors:** Nasima Frotan Yosufi, Bibi Sarah Yousofzai, Muhammad Subhan, Emran Yosufi

**Affiliations:** 1 Obstetrics and Gynecology, Rabia Balkhi Hospital, Kabul, AFG; 2 Obstetrics and Gynecology, Be Team International Cure Hospital, Kabul, AFG; 3 Department of Medicine, Allama Iqbal Medical College, Lahore, PAK; 4 Department of Medicine, Jinnah Hospital Lahore, Lahore, PAK; 5 Medicine and Surgery, Ghalib University, Kabul, AFG

**Keywords:** diabetes gestational, gestational hypertension, : hellp syndrome, hellp syndrome complications, liver hematoma, non-cardiogenic pulmonary edema, preeclampsia-eclampsia

## Abstract

Background

HELLP syndrome (Hemolysis, Elevated Liver Enzymes, and Low Platelets) is a severe obstetric complication associated with significant maternal morbidity and mortality. While its epidemiology is well-described in high-resource settings, data from conflict-affected and resource-limited regions like Afghanistan are critically scarce. This study aimed to delineate the incidence, risk profiles, complications, and outcomes of HELLP syndrome at a major tertiary healthcare hospital.

Methodology

A retrospective, descriptive case series was conducted at Rabia Balkhi Hospital in Kabul, reviewing all obstetric admissions from January 1 to December 31, 2022. Among 29,633 admissions, 26 patients met the inclusion criteria for HELLP syndrome based on established laboratory parameters (LDH >600 U/L, AST >70 U/L, platelets <100,000/mm³). Data on demographics, clinical presentation, risk factors, complications, and maternal outcomes were extracted and analyzed using descriptive statistics.

Results

The incidence of HELLP syndrome was 26 cases (0.09%) of all obstetric admissions. The most prevalent risk factors were abruptio placentae (10, 38.5%), diabetes mellitus (5, 19.2%), and chronic hypertension (4, 15.4%). Major maternal complications included disseminated intravascular coagulation (5, 19.2%), renal failure (4, 15.4%), and pulmonary edema (3, 11.5%). Notably, subcapsular liver hematoma, a rare but often fatal complication, was present in two cases (7.7%). The maternal mortality rate was 7.7% (*n* = 2), with a 92.3% recovery rate among survivors.

Conclusions

This study provides the first detailed epidemiological profile of HELLP syndrome. While the incidence aligns with some regional reports, the high prevalence of severe complications and a substantially elevated maternal mortality rate highlight critical systemic challenges. These findings underscore the urgent need for enhanced antenatal screening, early diagnosis, and improved access to critical care resources, including platelet transfusions and emergency surgical intervention, to mitigate preventable deaths in low-resource settings.

## Introduction

HELLP syndrome refers to a potential obstetric complication that presents with features like hemolysis, increased liver enzymes, and reduction in platelets [1]. The first description of this syndrome was given by Dr. Louis Weinstein in 1982 [1]. Since then, it has been established as a severe manifestation of preeclampsia and eclampsia, with an incidence of approximately 0.7% of pregnancies and up to 15% in patients who have severe preeclampsia or eclampsia [2]. It usually occurs in the third trimester or just after delivery [2]. It is often associated with nonspecific clinical symptoms, including epigastric or right upper quadrant abdominal pain, nausea, vomiting, headache, visual disturbances, fatigue, and malaise [2-3]. These symptoms may escalate rapidly, especially at night, making early recognition critical [2,3]. Its pathophysiology involves abnormal placental perfusion, endothelial dysfunction, platelet activation, and systemic inflammatory responses leading to microangiopathic hemolytic anemia, hepatic dysfunction, and thrombocytopenia [4]. Laboratory diagnostic criteria include a serum aspartate aminotransferase (AST) level >70 U/L, lactate dehydrogenase (LDH) >600 U/L, and platelet count <100,000/mm³ [3,4]. Hemolysis is confirmed through abnormal peripheral blood smears and elevated LDH levels, with total bilirubin >1.2 mg/dL [3,4]. Maternal complications include disseminated intravascular coagulation (DIC), abruptio placentae, renal and respiratory failure, and liver complications such as subcapsular hematoma and rupture (1%), with maternal death rates exceeding 50% in cases of hepatic rupture [5,6]. Life-threatening hepatic complications, especially in the presence of severe thrombocytopenia, demand prompt diagnosis and intervention [6]. Risk factors for liver rupture include advanced maternal age, multiparity, and severe preeclampsia [7].

Given its overlapping clinical features, HELLP syndrome must be differentiated from conditions such as acute fatty liver of pregnancy, glomerulonephritis, pyelonephritis, systemic lupus erythematosus, and thrombotic thrombocytopenic purpura [8]. CT scans, ultrasounds, and MRIs are imaging modalities used to diagnose hepatic hematomas [8]. In stable hemodynamic patients, conservative treatment may be attempted, whereas those with ruptured aneurysms require surgical intervention [9]. HELLP syndrome is curable only through prompt delivery, with vaginal birth preferred unless contraindicated by maternal or fetal conditions such as placental abruption or poor maternal status [10]. Management includes corticosteroids for fetal lung maturity if less than 34 weeks, magnesium sulfate for seizure prevention, and platelet transfusions for severe thrombocytopenia [11-13]. This study examined the incidence, clinical characteristics, risk factors, maternal complications, and outcomes of HELLP syndrome in 2022, aiming to strengthen evidence-based management in a resource-limited setting.

## Materials and methods

Study design and setting

A cross-sectional study was conducted at Rabia Balkhi Hospital in Kabul. The study reviewed all obstetric admissions over one year from January 1 to December 31, 2022.

Study population and demographics

The study included all obstetric patients admitted during the study period, yielding a total sample size of 29,633 obstetric admissions. Of these, 26 patients fulfilled the diagnostic criteria for HELLP syndrome and constituted the study sample. The HELLP syndrome cohort (*n* = 26) had a mean maternal age of 28.7 ± 4.2 years. Regarding obstetric history, 11 (42.3%) patients were nulliparous, while 15 (57.7%) were multiparous.

Inclusion and exclusion criteria

Patients were included if they were pregnant females diagnosed with HELLP syndrome based on standardized laboratory criteria consistent with the Tennessee classification system: evidence of hemolysis (lactate dehydrogenase >600 U/L), elevated liver enzymes (aspartate aminotransferase >70 U/L), and thrombocytopenia (platelet count <100,000/mm³) in the presence of clinically diagnosed preeclampsia or eclampsia [11].

Exclusion criteria included patients with incomplete medical records lacking essential laboratory parameters required to confirm HELLP syndrome, as well as those with alternative diagnoses or overlapping clinical syndromes, such as acute fatty liver of pregnancy, thrombotic thrombocytopenic purpura, hemolytic uremic syndrome, viral or autoimmune hepatitis, and severe preeclampsia without laboratory evidence of HELLP-when a definitive diagnosis of HELLP syndrome could not be established.

Data collection procedure

Data were collected retrospectively using a structured proforma. All primary data were obtained from paper-based patient medical records. Extracted variables included maternal age, parity, gestational age at presentation (recorded in weeks), clinical symptoms (including epigastric or right upper quadrant pain, nausea/vomiting, headache, and visual disturbances), documented risk factors, maternal complications, and final outcomes.

Diagnoses of risk factors and complications were confirmed by cross-referencing discharge summaries with laboratory reports and consultant notes. Data regarding intensive care unit (ICU) admission and duration of hospital stay were obtained from nursing logs, ward transfer records, and ICU admission registers.

For cases with incomplete documentation, the principal investigator conducted structured interviews with the attending obstetricians and senior residents to verify the diagnosis and confirm core outcome variables, including maternal survival, ICU admission, and major complications. No patient-reported data were collected directly from patients. Cases in which essential laboratory parameters or final maternal outcomes could not be reliably verified were excluded according to the study protocol.

Data sources

Data sources included paper-based hospital case files, laboratory reports, discharge summaries, operative notes, nursing and ward transfer records, ICU admission logs, and structured interviews with attending clinical staff solely for data verification purposes.

Statistical analysis

All data were analyzed using Microsoft Excel. Descriptive statistics, including frequencies and percentages, were employed for categorical variables. Due to the small sample size, inferential statistics were not applied. Results are presented in tabular format for clarity.

## Results

Incidence

Out of 29,633 obstetric admissions in 2022, 26 patients were diagnosed with HELLP syndrome, resulting in a prevalence of 0.09% (Table 1).

**Table 1:** Incidence of HELLP syndrome at Rabia Balkhi Hospital, 2022. HELLP, Hemolysis, Elevated Liver Enzymes, Low Platelets

Indicator	Number	Percentage
Patients with HELLP syndrome	26	0.09%
Patients with other obstetric conditions	29,607	99.91%
Total	29,633	100%

Clinical presentation and gestational age

At presentation, all patients (100%) had hypertension or features of preeclampsia. The most common presenting symptoms were epigastric or right upper quadrant pain (18, 69.2%), nausea or vomiting (14, 53.8%), and headache or visual disturbances (12, 46.2%).

Most patients presented in the late third trimester, with an estimated gestational age ranging from 34 to 39 weeks at diagnosis.

Demographics

The demographic characteristics, clinical presentation, and documented risk factors of the 26 patients diagnosed with HELLP syndrome are summarized in Table 2.

**Table 2:** Demographic characteristics, clinical presentation, and risk factors of patients with HELLP syndrome. Percentages are calculated based on the total cohort (*n* = 26). HELLP syndrome diagnosis was based on standard laboratory criteria (LDH >600 U/L, AST >70 U/L, platelets <100,000/mm³). RUQ, right upper quadrant; LDH, lactate dehydrogenase; AST, aspartate aminotransferase; HELLP, Hemolysis, Elevated Liver Enzymes, and Low Platelets

Variable category	Parameter	Number (*n*)	Percentage (%)
Demographics	Nulliparous	11	42.3
	Multiparous	15	57.7
Clinical presentation	Hypertension/preeclampsia features	26	100
	Epigastric/RUQ pain	18	69.2
	Nausea/vomiting	14	53.8
	Headache/visual symptoms	12	46.2
Documented risk factors	Abruptio placentae	10	38.5
	Diabetes mellitus (type 2)	5	19.2
	Chronic hypertension	4	15.4
	Chronic kidney disease	4	15.4
	Anemia	3	11.5

Risk factors

**Figure 1:**
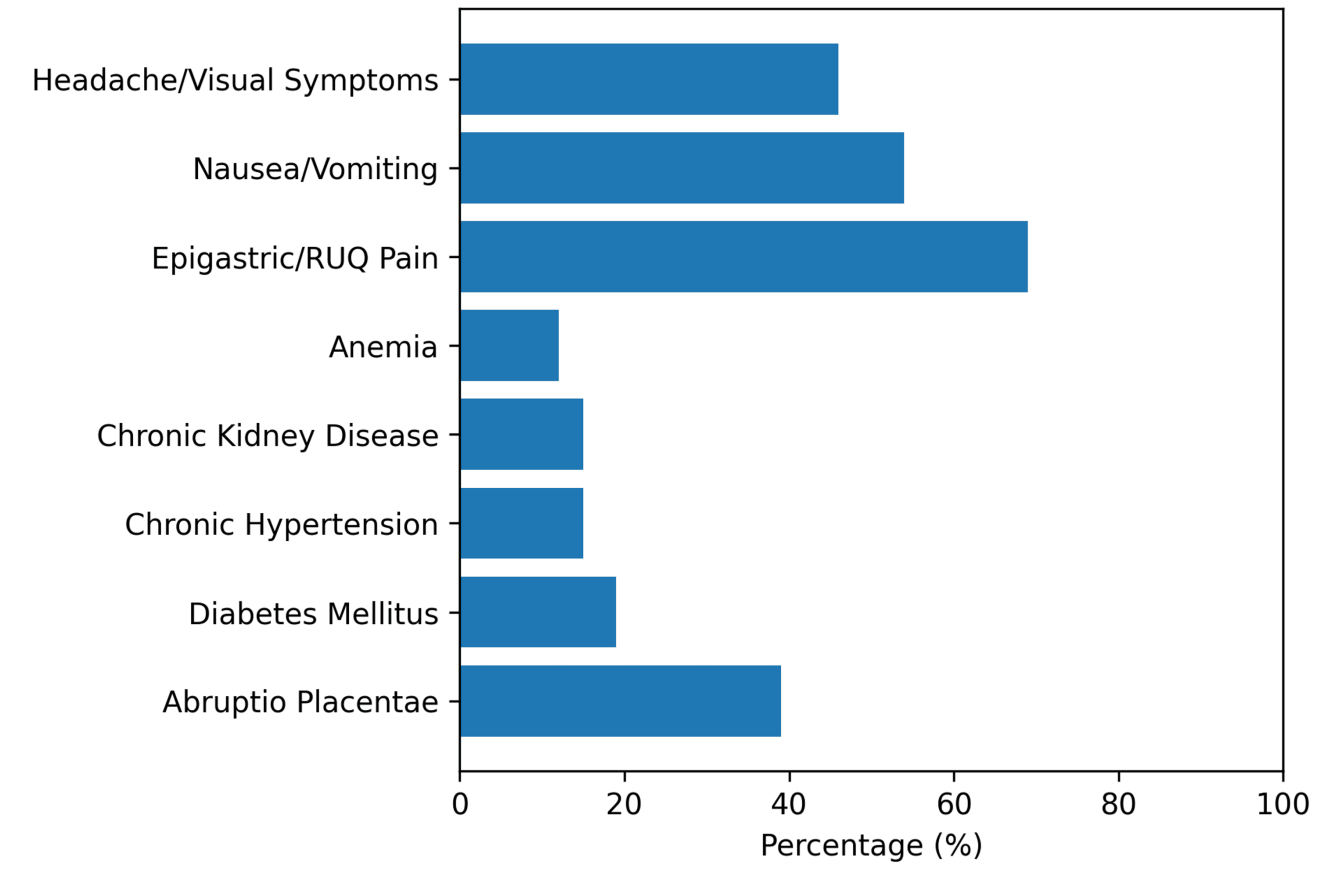
Risk factors and clinical presentation among patients diagnosed with HELLP syndrome at Rabia Balkhi Hospital (n = 26). Most patients presented in the late third trimester (approximately 36-39 weeks of gestation). Percentages are calculated from the total HELLP cohort. HELLP, Hemolysis, Elevated Liver Enzymes, and Low Platelets

Among the 26 HELLP syndrome cases, 10 (39%) had abruptio placentae, 5 (19%) had type 2 diabetes mellitus, 4 (15%) had chronic hypertension, 4 (15%) had chronic kidney disease, and 3 (12%) had anemia. These clinical features and risk factors are summarized in Figure 1.

Complications

**Figure 2:**
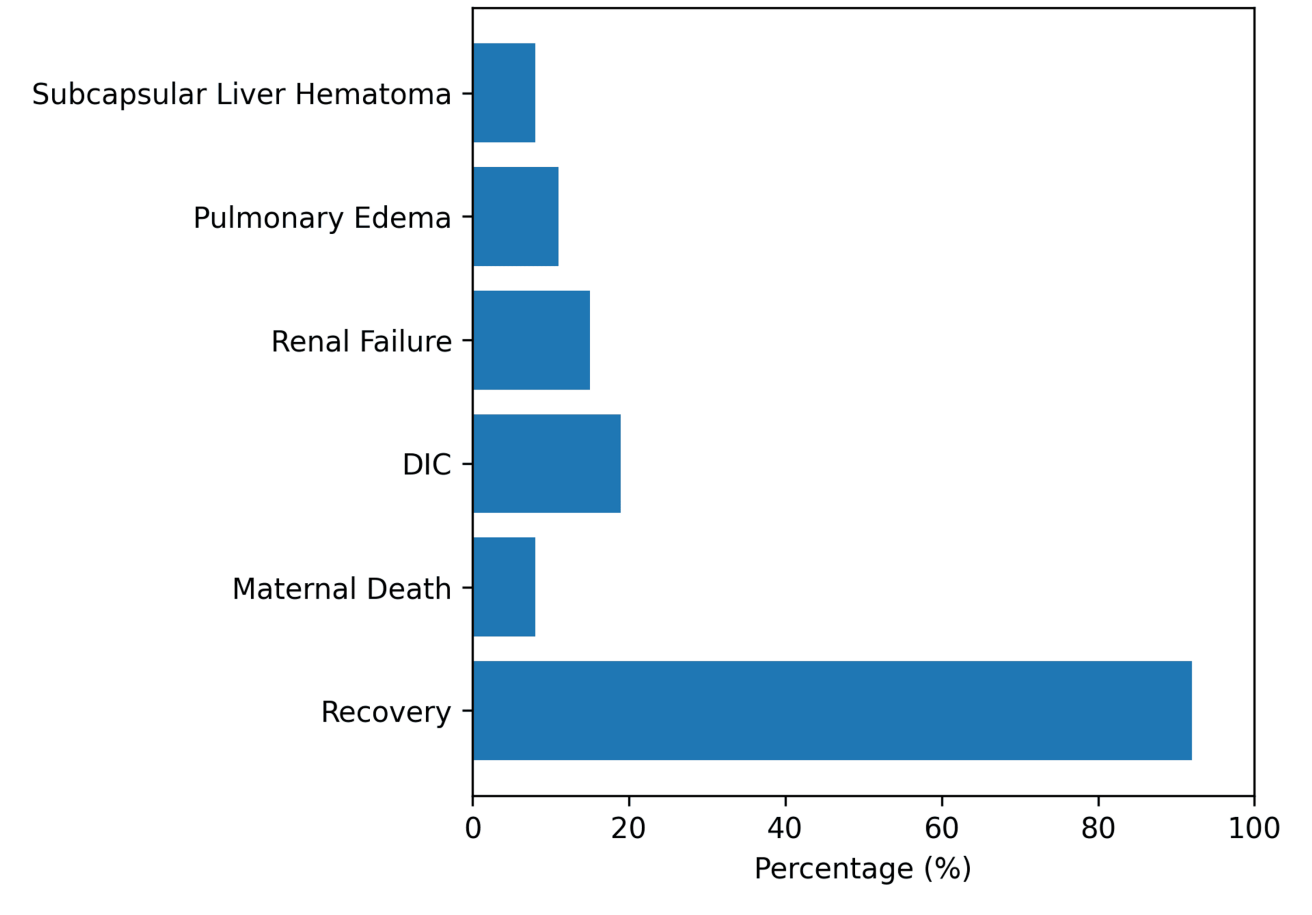
Maternal complications and outcomes in patients with HELLP syndrome at Rabia Balkhi Hospital (n = 26). Recovery occurred in 24 (92.3%) cases, while maternal mortality was observed in two cases (7.7%). HELLP, Hemolysis, Elevated Liver Enzymes, and Low Platelets

Maternal complications were observed in a substantial proportion of patients diagnosed with HELLP syndrome. The most frequently encountered complication was abruptio placentae, occurring in 10 (38.5%) patients. Disseminated intravascular coagulation occurred in 5 (19.2%), renal failure in 4 (15.4%), pulmonary edema in 3 (11.5%), and subcapsular liver hematoma in 2 (7.7%). A total of 9 (34.6%) patients required admission to the ICU. The median duration of hospital stay ranged from 5 to 12 days, with longer stays observed among patients with multiorgan complications. Overall, maternal outcomes were favorable, with 24 (92.3%) patients recovering. Maternal mortality occurred in 2 (7.7%) patients. The distribution of maternal complications and outcomes is illustrated in Figure 2.

## Discussion

This study investigated the prevalence, clinical features, risk factors, complications, and maternal outcomes of HELLP syndrome at a tertiary facility. Results were consistent with earlier studies, albeit demonstrating notable differences compared to other regional clinical settings and patient populations. The prevalence of HELLP syndrome in this study was 0.09% (26 out of 29,633 obstetric admissions), aligning closely with the 0.1% prevalence reported in a Bangladeshi survey conducted among the general obstetric population [12]. However, this prevalence was considerably lower than that reported in a Pakistani study, where 2.8% (15 out of 621) of patients with eclampsia developed HELLP syndrome [13]. These differences may be attributed to variations in the populations studied, diagnostic efforts, levels of clinical training, and the availability of resources, whether limited or abundant [12,13]. In addition, single-center data are likely to misrepresent broader epidemiological trends due to referral and admission biases [12,13].

In the present study, the most common risk factors for HELLP syndrome were abruptio placentae (10, 39%), type 2 diabetes mellitus (5, 19%), chronic hypertension (4, 15%), chronic kidney disease (4, 15%), and anemia (3, 12%). These findings are consistent with those reported in the Bangladeshi and Pakistani studies [12,13]. In Bangladesh, chronic kidney disease (14%), liver failure (15%), DIC (23%), placental abruption (38%), and diabetes (18%) were the most frequently reported contributors [12], while the Pakistani study reported slightly higher proportions for diabetes (22%) and chronic kidney disease (16%), with comparable rates of placental abruption (36%) [13]. Although subtle differences may exist in the exact etiological mechanisms, the consistent reporting of these risk factors across studies underscores their pathophysiological relevance [3,4,5]. Placental abruption is believed to induce placental ischemia and release pro-inflammatory mediators [8], whereas diabetes and hypertension may compromise endothelial function, accelerating hemolysis and platelet consumption [3,4].

The most prevalent complications observed in this study were abruptio placentae (10, 39%), DIC (5, 19%), renal failure (4, 15%), pulmonary edema (3, 11%), and subcapsular liver hematoma (2, 8%). These findings diverge somewhat from the Pakistani study, which reported lower rates of pulmonary edema (6%) and liver hematoma (1%) [13]. The high frequency of severe complications in our cohort could be due to delayed referrals, inadequate antenatal screening, or limited access to intensive care in low-resource settings [12,13]. Although overall recovery rates remain encouraging, the observed maternal mortality highlights the need for enhanced diagnostic infrastructure, critical care capacity, and early multidisciplinary interventions [11]. The two maternal deaths likely occurred due to advanced disease at presentation, despite treatment. In this study, maternal recovery was 92%, with a mortality rate of 8% (2 out of 26), compared to 2% and 3% mortality rates reported in Bangladesh and Pakistan, respectively [12,13]. The rare occurrence of subcapsular liver hematoma is notable due to its association with high maternal mortality [6].

A prospective cross-sectional study from Uganda recently reported a remarkably high prevalence of HELLP syndrome at 18.6% among women with preeclampsia/eclampsia (24/129, 95% confidence interval (CI) 12.7-26.3%) using standard Tennessee criteria, substantially higher than our findings [13]. Factors independently associated with HELLP included maternal age under 20 years (adjusted prevalence ratio (aPR) 4.96) and epigastric pain (aPR 5.89), highlighting the importance of demographic and symptomatic markers for early detection [13]. Additionally, a recent Cochrane-backed systematic review on corticosteroid therapy in HELLP syndrome concluded that evidence remains inconclusive [11]. Maternal mortality risk ratios ranged widely (RR 0.77, 95% CI 0.25-2.38), and effects on renal failure and liver complications were uncertain [11]. Although steroids may transiently improve hematologic parameters, they have not demonstrated clear long-term benefits on patient-important clinical outcomes [11].

Increased monitoring and prompt imaging for liver rupture should be considered, especially in patients with advanced maternal age, multiparity, and severe preeclampsia presenting with upper quadrant pain or peritoneal signs [7]. Delayed presentation and limited access to corticosteroids, platelet transfusions, and emergency operative interventions likely contribute to adverse outcomes [11]. Limitations of this retrospective, single-center study include reliance on paper-based medical records, incomplete documentation in some cases, and the absence of long-term maternal and neonatal follow-up. Verification through clinician interviews was required for a minority of cases, which may introduce recall bias, although this was limited to confirmation of objective outcomes [1,2]. Nonetheless, this study provides critical clinical insight into HELLP syndrome in a resource-constrained environment [1,2]. Timely identification and management of high-risk pregnancies, particularly those complicated by preeclampsia or abruptio placentae, remain essential [1,2]. Outcomes can be improved through enhanced healthcare worker training, better diagnostic capacity, and timely interventions, including corticosteroids and transfusions [11]. Future multicenter prospective studies are needed to develop standardized care protocols tailored to healthcare contexts [12,13].

## Conclusions

HELLP syndrome remains a critical obstetric emergency with significant maternal risk, particularly in resource-limited settings. Early recognition through vigilant clinical and laboratory monitoring, especially among high-risk pregnancies, is essential. Optimizing outcomes requires comprehensive antenatal care, timely diagnosis, and coordinated multidisciplinary management, supported by access to corticosteroids, transfusion services, and emergency obstetric interventions. Despite the limitations inherent to a single-center, retrospective study, these findings provide valuable context for improving maternal care and underscore the need for multicenter research to develop standardized, context-specific management protocols.
